# Anti-Sclerostin Antibody Inhibits Internalization of Sclerostin and Sclerostin-Mediated Antagonism of Wnt/LRP6 Signaling

**DOI:** 10.1371/journal.pone.0062295

**Published:** 2013-04-29

**Authors:** Maarten van Dinther, Juan Zhang, Stella E. Weidauer, Verena Boschert, Eva-Maria Muth, Achim Knappik, David J. J. de Gorter, Puck B. van Kasteren, Christian Frisch, Thomas D. Mueller, Peter ten Dijke

**Affiliations:** 1 Department of Molecular Cell Biology and Centre for Biomedical Genetics, Leiden University Medical Center, Leiden, The Netherlands; 2 Department of Molecular Plant Physiology and Biophysics, Julius-von-Sachs Institut, Wuerzburg, Germany; 3 AbD Serotec, a Bio-Rad company, Puchheim, Germany; Georgia Regents University, United States of America

## Abstract

Sclerosteosis is a rare high bone mass disease that is caused by inactivating mutations in the *SOST* gene. Its gene product, Sclerostin, is a key negative regulator of bone formation and might therefore serve as a target for the anabolic treatment of osteoporosis. The exact molecular mechanism by which Sclerostin exerts its antagonistic effects on Wnt signaling in bone forming osteoblasts remains unclear. Here we show that Wnt3a-induced transcriptional responses and induction of alkaline phosphatase activity, an early marker of osteoblast differentiation, require the Wnt co-receptors LRP5 and LRP6. Unlike Dickkopf1 (DKK1), Sclerostin does not inhibit Wnt-3a-induced phosphorylation of LRP5 at serine 1503 or LRP6 at serine 1490. Affinity labeling of cell surface proteins with [^125^I]Sclerostin identified LRP6 as the main specific Sclerostin receptor in multiple mesenchymal cell lines. When cells were challenged with Sclerostin fused to recombinant green fluorescent protein (GFP) this was internalized, likely via a Clathrin-dependent process, and subsequently degraded in a temperature and proteasome-dependent manner. Ectopic expression of LRP6 greatly enhanced binding and cellular uptake of Sclerostin-GFP, which was reduced by the addition of an excess of non-GFP-fused Sclerostin. Finally, an anti-Sclerostin antibody inhibited the internalization of Sclerostin-GFP and binding of Sclerostin to LRP6. Moreover, this antibody attenuated the antagonistic activity of Sclerostin on canonical Wnt-induced responses.

## Introduction

The mass, biomechanical properties and structural integrity of bone is kept in balance by continuous cycles of bone resorption and bone formation [1], [2]. In osteoporosis, the balance between bone degradation and formation is perturbed: more bone is broken down than is formed [3]. Osteoporosis has a high incidence and patients can, amongst others, be treated with bisphosphonates, selective estrogen modulators and inhibitors of RANKL [4], all of which can effectively prevent further bone loss. However, since osteoporosis is often diagnosed at a stage when extensive bone loss has already occurred, there is a dire need for novel therapies that stimulate new bone formation to restore bone integrity [5]. Whereas osteoporosis is defined by an overall bone loss, on the other side of the spectrum are rare diseases that are characterized by excessive bone formation [6], [7]. In contrast to the multi-factorial osteoporosis, the high bone mass disorders are often monogenic. The genes that are linked to these disorders are considered to be potential therapeutic targets for the treatment of osteoporosis [8].

One example of a high bone mass disease is Sclerosteosis, which affects a number of families in South Africa [9], [10]. This disease has been linked to mutations in the *SOST* gene that lead to inactivation of its product Sclerostin [11], [12]. The absence of this protein leads to dramatic bone overgrowth in mice and overactivity of canonical Wnt signaling in bone tissue [13], [14]. Sclerostin is expressed and subsequently secreted by osteocytes [10], [15] and interacts with the Wnt co-receptors low density lipoprotein receptor-related protein (LRP) 5 and 6 [16]–[18]. These are single transmembrane proteins that share 73% sequence identity and are essential for canonical Wnt signaling [19], [20]. Both contain in their extracellular domain four six-bladed β-propeller structures with so-called YWTD repeats. The four propellers share only 19% sequence similarity among each other and have different functional properties. Sclerostin was shown to interact with the first, most amino-terminal propellers of both LRP5 and 6 [21]. Interestingly, gain of function mutations in LRP5 result in high bone mass [22], [23]. These gain of function LRP5 mutants show reduced Sclerostin binding [24]. Sclerostin has recently been shown to also interact with LRP4 and certain mutations in this receptor were found to decrease the interaction with Sclerostin [25].

Canonical Wnt signaling is initiated by direct binding and heteromeric complex formation of seven-transmembrane receptor Frizzled proteins and the LRP5 and 6 co-receptors upon interaction with specific Wnt ligands, which leads to the stabilization of cytoplasmic β-Catenin [26]. In the absence of Wnt ligands, β-Catenin forms a complex that includes Adenomatous polyposis coli (APC), Axin and Glycogen synthase kinase 3 (GSK3). This complex facilitates phosphorylation and subsequent proteasomal degradation of β-Catenin. In the presence of Wnt ligands, this complex dissociates, and β-Catenin accumulates and translocates to the nucleus, where it interacts with TCF/Lef1 transcription factors and initiates transcription of specific target genes, such as Axin [26], [27].

Like Sclerostin, Dickkopf 1 (DKK1) glycoproteins inhibit canonical Wnt signaling by binding to LRP5 and 6 [28]. DKK1 mainly interacts with the third and fourth propeller of these proteins [29], but can also bind to the first and second propellers [29], [30]. At least two mechanisms have been proposed by which DKK1 exerts its antagonistic effects on LRP5 and 6: DKK1 mediates the recruitment of co-receptor Kremen to LRP5 and 6, thereby inducing endocytosis of LRP5 and 6 [28], [31] and/or DKK1 disrupts the formation of the Wnt-induced Frizzled-LRP6 complex [32].

Here we describe the genetic and biochemical interaction of Sclerostin with the Wnt co-receptors LRP5 and LRP6. In addition, we show that GFP-tagged Sclerostin is internalized, most likely via a Clathrin dependent pathway, and is subsequently degraded in a proteasome-dependent manner. Moreover, we describe antibodies that specifically interfere with binding of Sclerostin to Wnt co-receptors and stimulate osteoblast differentiation. Such neutralizing Sclerostin antibodies may be used for future anabolic treatment of osteoporosis.

## Results

### Wnt/β-catenin-induced Responses Depend on LRP5 and LRP6

**Figure 1:**
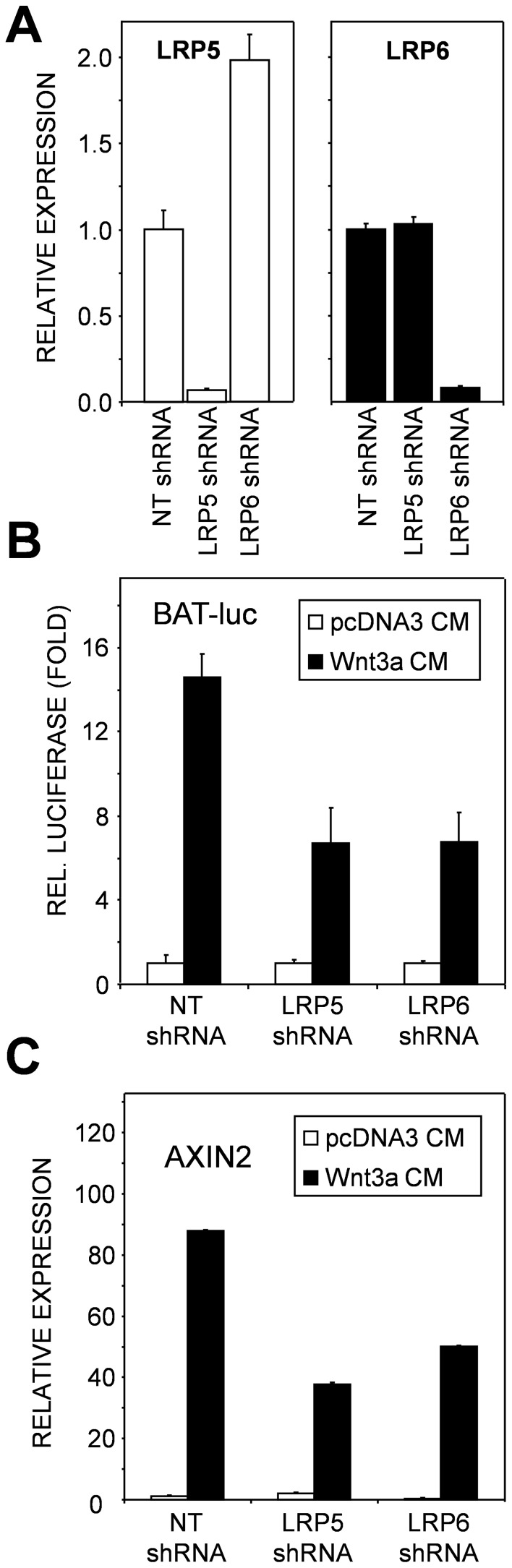
Small hairpin RNA (shRNA) mediated knockdown of LRP5 and LRP6 inhibits Wnt3a-induced responses. (A) qRT-PCR was performed on cDNA obtained from C2C12 cells with stable knockdown of LRP5 and LRP6 to determine the efficiency of the knockdown. (B) C2C12 cells with stable knockdown of LRP5 and LRP6 were transiently transfected with the BAT-luc transcriptional reporter construct and stimulated with control (pcDNA CM) or Wnt3a conditioned media (Wnt3a CM). Luciferase activity was determined 16 h after stimulation. (C) C2C12 cells with stable knockdown of LRP5 and LRP6 were stimulated with the indicated conditioned media. After 16 h of stimulation RNA was isolated and AXIN2 mRNA levels were determined by qRT-PCR.

To investigate the role of LRP5 and LRP6 in Wnt-induced responses, Wnt3a-conditioned media was used to stimulate the mouse myoblast cell line C2C12 cells, which were depleted for either LRP5 or LRP6 by lentiviral shRNA mediated knockdown. C2C12 cells transduced with a non-targeting shRNA construct served as a control. Knockdown efficiency was determined by quantitative real-time PCR (qRT-PCR) and both LRP5 and LRP6 were efficiently and specifically targeted by their specific shRNAs. Knockdown of LRP6 induced some up-regulation of LRP5 mRNA expression. (Fig. 1a). Knockdown of LRP5 or LRP6 was equally efficient in inhibiting the Wnt3a-conditioned media induced canonical signaling as measured by β-Catenin/TCF-dependent BAT-luc transcriptional luciferase reporter activity (Fig. 1b) and induction of mRNA expression of AXIN2, a direct Wnt target gene (Fig. 1c). Apparently, the LRP6 up-regulation in LRP5 knockdown cells was insufficient to obtain a rescue of the response. When Wnt3a conditioned media was replaced with recombinant Wnt3a similar results were obtained (Fig. S1a). Thus both co-receptors are critically important in the Wnt/β-catenin responses in C2C12 cells. Similar results were obtained when cells of the osteoprogentior cell line KS483 were depleted of LRP5 or LRP6 by shRNA or siRNA (data not shown and [33]). When using another activator of canonical Wnt signaling (recombinant Wnt9b) there was a weak induction of AXIN2 mRNA expression. Whereas this response was strongly inhibited by LRP5 knockdown, it was only moderately inhibited by LRP6 depletion (Fig. S1b).

### Sclerostin Inhibits Wnt-induced Osteoblast Differentiation, but not LRP5 and LRP6 Phosphorylation

**Figure 2:**
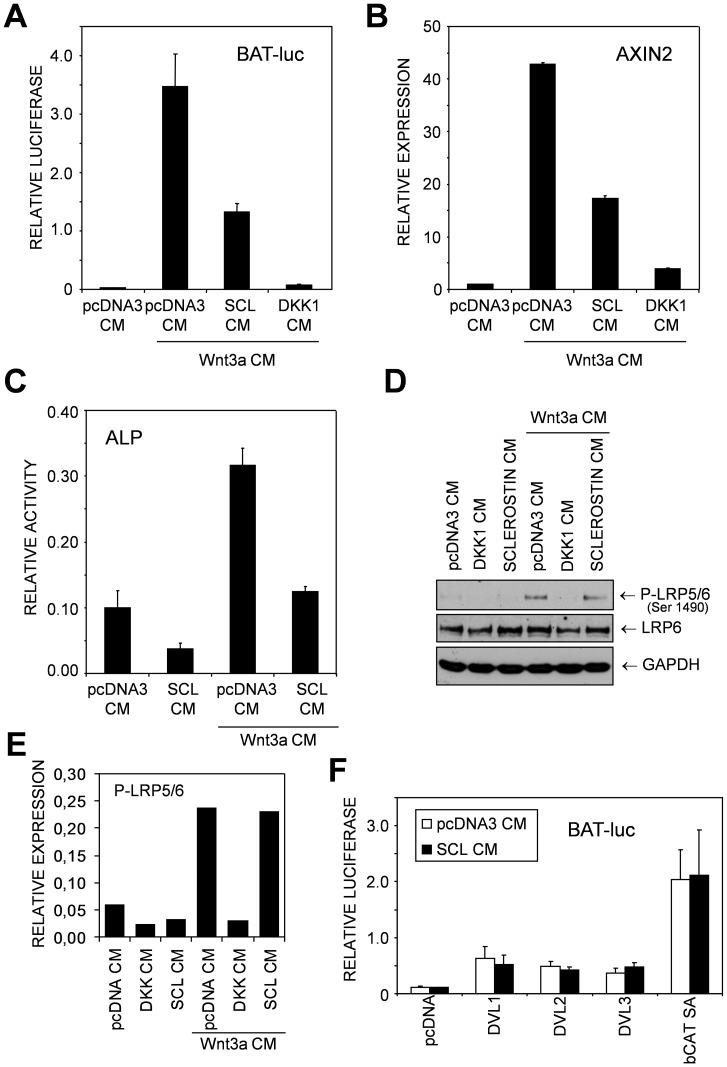
Sclerostin inhibits Wnt3a-induced osteoblast differentiation, but not LRP5/6 phosphorylation. (A) KS483 cells were transiently transfected with the BAT-luc reporter construct. The cells were stimulated with conditioned control media (pcDNA CM) or Wnt3a conditioned media (Wnt3a CM), in the presence of Sclerostin conditioned media (SCL CM) or Dickkopf 1 conditioned media (DKK1 CM). Luciferase activity was determined 16 h after stimulation. (B) KS483 cells were stimulated with the indicated conditioned media and after 16 h RNA was isolated and AXIN2 mRNA levels were determined by qRT-PCR. (C) KS483 cells were stimulated with the indicated conditioned media and after three days the cells were fixed and stained for ALP activity. Staining was dissolved and measured spectrophotometrically. (D) KS483 cells were stimulated with the indicated conditioned media, next the cells were lysed and Western blot was performed. (E) Phospho-LRP5/6 bands were quantified and normalised to the LRP6 bands. (F) KS483 cells were transiently transfected with the BAT-luc reporter construct together with the indicated plasmids (Dvl1, Dvl2, Dvl3 or bCAT-SA). The cells were stimulated with conditioned control media or Sclerostin conditioned media. Luciferase activity was determined 16 h after stimulation.

Consistent with previous reports [10], [16], [17], we found that Sclerostin-containing conditioned media (Sclerostin-CM; SCL-CM) inhibited Wnt-induced BAT-luc transcriptional reporter activation (Fig. 2a) and Wnt3a-induced AXIN2 mRNA transcription (Fig. 2b) in the osteoprogenitor cell line KS483. Wnt-induced Alkaline Phosphatase (ALP) activity, an early marker for osteoblast differentiation, was also inhibited by Sclerostin-CM (Fig. 2c). Similar results were obtained using recombinant murine Sclerostin instead of Sclerostin-CM (data not shown).

After stimulation with Wnt3a, LRP6 is phosphorylated, which results in stabilization of β-Catenin [34]. In contrast to the classical Wnt antagonist DKK1, Sclerostin did not inhibit Wnt3a-induced phosphorylation of LRP6 on serine 1490 in the first PPSPXS motif (Fig. 2d and 2e). Similar results were obtained for Wnt3a-induced LRP5 phosphorylation (data no shown), suggesting that Sclerostin acts downstream of LRP5/6. To activate Wnt signaling responses without activating LRP5/6 we used ectopic expression of Dishevelled 1 (DVL1), Dishevelled 2 (DVL2), Dishevelled 3 (DVL3) and a degradation insensitive β-Catenin mutant (β-CAT SA). When over-expressed these proteins activate the BAT-luc reporter, but none of these activations could be blocked by Sclerostin (Fig. 2f). Thus, the mechanism of action of LRP5/6 inhibition by Sclerostin appears to be different than by DKK1.

### Sclerostin Differentially Interacts with LRP5 and LRP6

**Figure 3:**
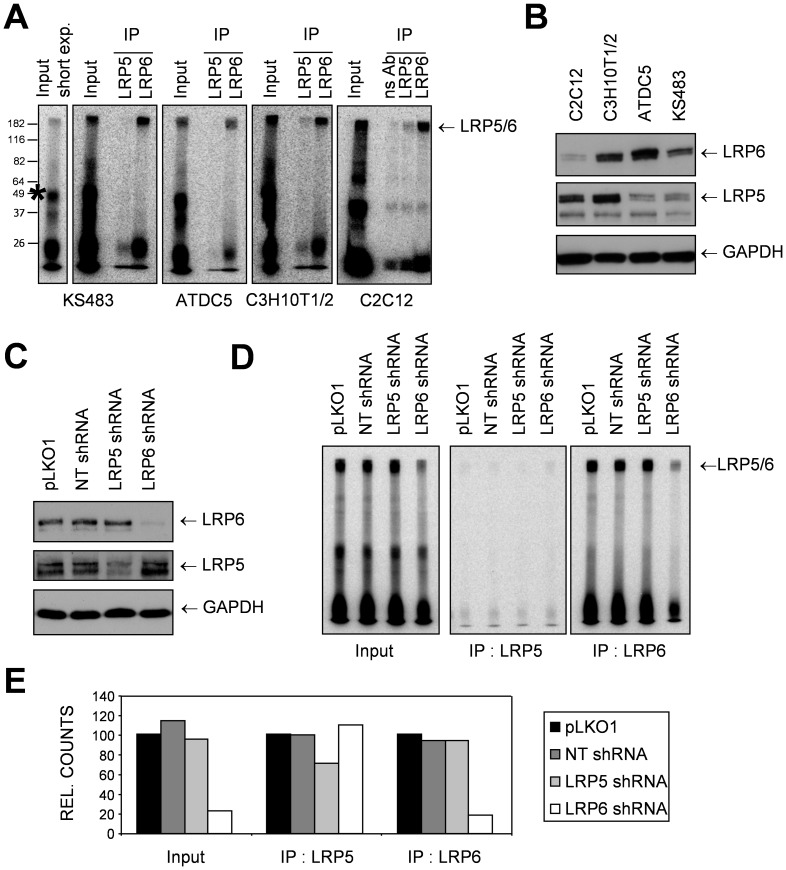
Sclerostin differentially interacts with LRP5/6. (A) C2C12, C3H10T1/2, ATDC5 and KS483 were affinity labeled using [^125^I]Sclerostin, followed by chemical crosslinking. Cells were lysed and an immunoprecipitation was performed using LRP5 and LRP6 antibodies. Samples were separated on a SDS-PAGE gel and the radioactive signal was visualized using a phosphorimager screen. The unknown 50 kD band is marked with an asteriks on the short exposure of the KS483 input. (B) C2C12, C3H10T1/2, ATDC5 and KS483 cells were lysed and Western blot was performed to determine the expression of LRP5 and LRP6, GAPDH was used as a loading control. (C) Lysates were made of KS483 control cells (pLKO and NT-shRNA) and KS483 cells with stable LRP5 or LRP6 knockdown. Knockdown efficiency was determined by Western blotting. (D) KS483 control cells and KS483 cells with stable LRP5 or LRP6 knockdown were affinity labeled using [^125^I]Sclerostin, followed by chemical crosslinking. Cells were lysed and an immunoprecipitation was performed using LRP5 and LRP6 antibodies. LRP5 and LRP6 antibodies do not cross-react (Fig. S1a). Samples were separated on a SDS-PAGE gel and the radioactive signal was visualized using a phosphorimager screen. (E) High molecular weight bands corresponding to LRP5 and LRP6 were quantified using ImageQuant TL v2003.03 software (Amersham Biosciences), and normalized to the pLKO control.

To gain more insight into the interaction of Sclerostin with LRP5 and LRP6, we radiolabelled recombinant murine Sclerostin using ^125^Iodine. Sclerostin-interacting cell surface proteins of different cell lines were affinity-labeled with [^125^I]Sclerostin, upon which the formed complexes were crosslinked using Bis(Sulfosuccinimidyl) suberate (BS3) and Disuccinimidyl suberate (DSS). Subsequently, the complexes containing LRP5 and LRP6 were immunoprecipitated using LRP5 or LRP6 specific antibodies. Specificity of these anti-LRP5 and anti-LRP6 antibodies was tested in COS-1 cells over-expressing tagged LRP5 and LRP6; no cross-reactivity was observed (Fig. S2a). Samples were separated by SDS-PAGE and the radioactive signal was visualized using a phosphorimager screen. Sclerostin was found to bind strongly to LRP6 and weakly to LRP5 (Fig. 3a). The expression of LRP5 and LRP6 was measured by Western blot and qRT-PCR. The mRNA expression levels were similar to the protein expression with apparent higher levels of LRP5 and -6 transcription in C3H10T1/2 cells than in other examined cell types (Fig. 3b, S2b and S2c). In all the examined cell types recombinant Wnt3a induced AXIN2 mRNA expression, and Sclerostin attenuated this expression. No apparent relation could be observed between the expression of LRP5 or LRP6 and the level of inhibition (Fig. S2d). In the crosslinking experiments a very strong band was observed with an apparent size of approximately 50 kDa (Fig. 3a, asterisks), which was not immunoprecipitated using LRP5 or LRP6 specific antibodies. This suggests a Sclerostin binding partner with a molecular weight of around 25 kDa which is not in complex with LRP5/6, and may actually be Sclerostin itself. Sclerostin is a monomer in solution even at rather elevated concentrations [35], but may transiently form dimers under the conditions of the crosslinking procedure.

Using lentiviral shRNAs we created KS483 cell lines with LRP5 or LRP6 knockdown. Knockdown was confirmed by Western blotting (Fig. 3c). These KS483 knockdown lines were affinity labeled with [^125^I]Sclerostin, complexes were crosslinked and those containing LRP5 and LRP6 were immunoprecipitated. With the use of ImageQuant TL software (Amersham Biosciences) we quantified the radioactive signal. Whereas knockdown of LRP6 resulted in a strong reduction on the total binding of [^125^I]Sclerostin to the high molecular weight complexes (containing both LRP5 and LRP6), knockdown of LRP5 had little to no effect, consistent with LRP6 being the main interaction partner of Sclerostin in KS483 cells. In addition, knockdown of either LRP5 or LRP6 had no effect on binding of Sclerostin to the remaining LRP (Fig. 3d and 3e).

### Internalization of Sclerostin-GFP

**Figure 4:**
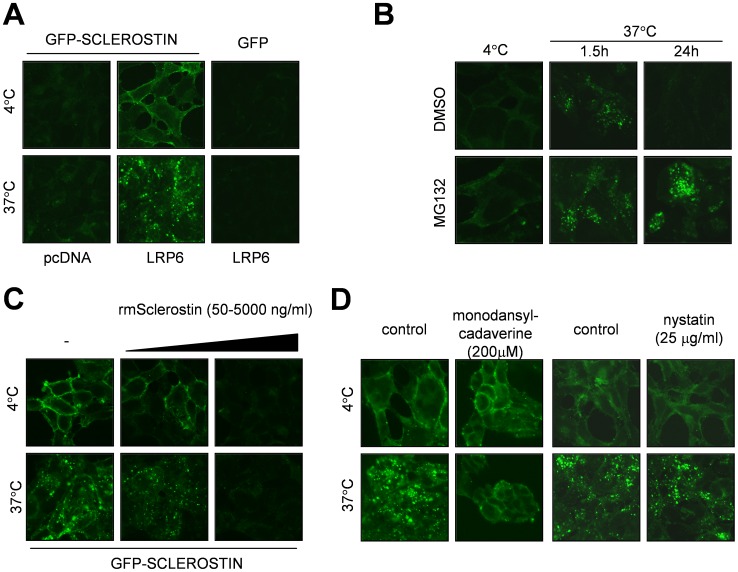
Internalization of Sclerostin-GFP. (A) 293 cells stably over-expressing LRP6 (293-LRP6) or containing an empty pcDNA3 vector, were incubated with GFP or Sclerostin-GFP for 1.5 hours at 4°C. After binding the cells were transferred to a 37°C incubator for 30 minutes, after this incubation the cells were fixed and confocal images were taken. (B) 293-LRP6 cells were first pre-treated with DMSO or MG132 (10 µM) for 4 hours, and then incubated with Sclerostin-GFP for 1.5 h at 4°C. After binding the cells were transferred to a 37°C incubator for the indicated time periods, next the cells were fixed and confocal images were taken. (C) 293-LRP6 cells were incubated with Sclerostin-GFP with different amounts of non-tagged Sclerostin for 1.5 h at 4°C. After binding the cells were transferred to a 37°C incubator for 30 minutes, after this incubation the cells were fixed and confocal images were taken. (D) 293-LRP6 cells were pre-treated with Monodansylcadaverine (200 µM) or Nystatin (25 µg/ml) or its relevant controls for 30 minutes. Next Sclerostin-GFP was added and the cells were incubated for 1.5 h at 4°C. After binding the cells were transferred to a 37°C incubator for 30 minutes, next the cells were fixed and confocal images were taken.

Based upon previous reports on the mechanism of action of DKK1 [36], we hypothesized that Sclerostin is internalized in a LRP5/6 dependent manner similar to DKK1. With LRP6 being the main receptor for Sclerostin, we stably transfected 293 cells with a plasmid encoding LRP6-V5 in order to study the binding and internalization of recombinant murine Sclerostin-GFP. LRP6 over-expressing cells were incubated on ice (to prevent internalization) with Sclerostin-GFP fusion protein or GFP as a negative control, after incubation for 1.5 h to allow for binding the cells were placed in a 37°C incubator for 30 min. Cells over-expressing LRP6 showed an increase of Sclerostin-GFP binding and internalization compared to control cells transfected with empty vector (Fig. 4a). Internalized Sclerostin-GFP was present as small puncta. When incubated for longer time at 37°C the Sclerostin-GFP protein was degraded in a proteasome-dependent manner, as degradation could be prevented by adding the proteasomal inhibitor MG132 (Fig. 4b). As expected, binding and internalization of Sclerostin-GFP could be competed away with an excess of non-tagged recombinant murine Sclerostin (Fig. 4c). Wild type Sclerostin was a highly effective competitor of Sclerostin-GFP binding to LRP6. The GFP-tag on Sclerostin may attenuate its apparent affinity for LRP6.

To investigate which kind of endocytosis route is employed by Sclerostin-GFP/LRP6 we used two inhibitors: Monodansylcadaverine *for inhibition of the Clathrin-dependent endocytosis pathway* [*37*] and Nystatin to inhibit the Caveolin-dependent endocytosis pathway [38]. Both inhibitors showed no effect on the binding of Sclerostin-GFP to LRP6 over-expressing cells. With respect to internalization, Monodansylcadaverine clearly showed inhibition of internalization, whereas Nystatin had no effect on internalization (Fig. 4d). Thus, these results suggest that Sclerostin-GFP is internalized via the Clathrin-mediated endocytosis pathway, similar as GFP-DKK1 ([36], and data not shown).

Binding and internalization of Sclerostin-GFP possibly to endogenous LRPs was also observed in KS483, albeit weaker than in LRP6 over-expressing cells (Fig. S2e). In these cells, knockdown of LRP5 or LRP6 had no apparent effect on binding and internalization of Sclerostin-GFP (Fig. S2f), suggesting that Sclerostin-GFP may also be internalized in a LRP5/6 independent manner in KS483 cells.

### Inhibition of Sclerostin Function by Neutralizing Antibodies

**Figure 5:**
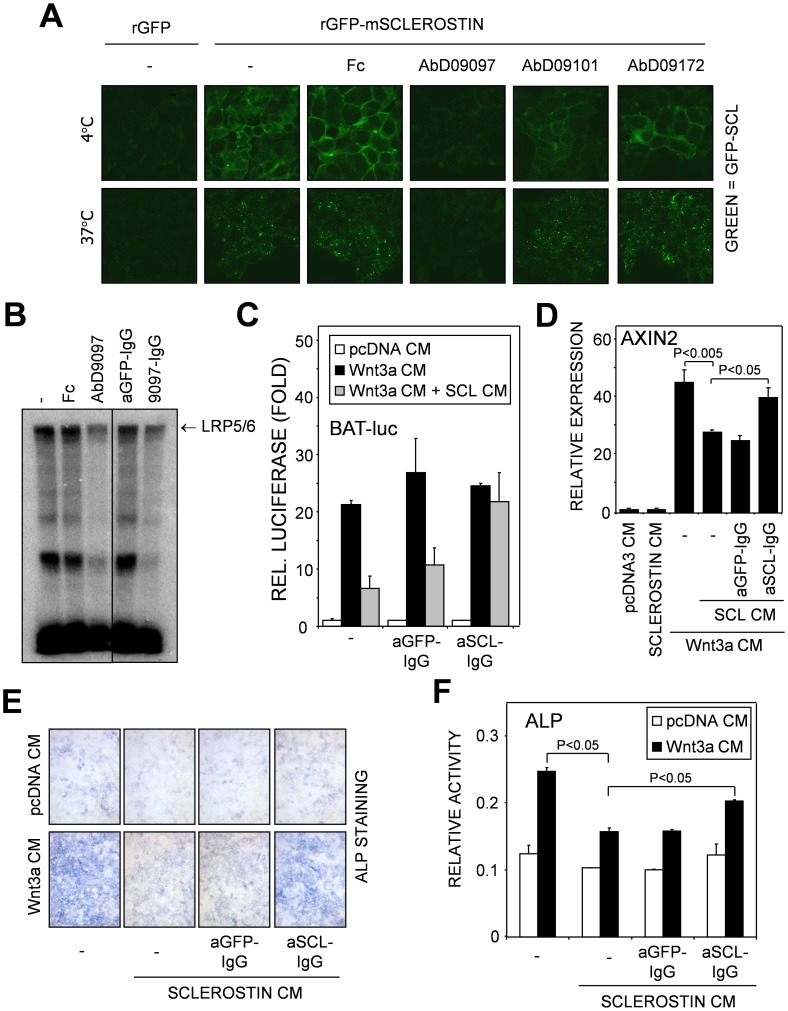
Inhibition of Sclerostin function by antibodies. (A) Sclerostin-GFP was pre-incubated with Fc or the different Fabs raised against Sclerostin for 30 minutes at room temperature, next the mixtures were added to 293-LRP6 cells for 1.5h at 4°C. After binding the cells were transferred to a 37°C incubator for 30 minutes, next the cells were fixed and confocal images were taken. (B) KS483 cells were affinity labeled with [^125^I]Sclerostin that was pre-incubated for 30 minutes at room temperature with controls (Fc and aGFP-IgG) or Sclerostin neutralizing antibodies (AbD9097 and aSCL-IgG), followed by chemical crosslinking. Cells were lysed and samples were separated by SDS-PAGE. The radioactive signal was visualized using a phosphorimager screen. (C) KS483 cells were transiently transfected with the BAT-luc reporter construct. The cells were stimulated with conditioned control media (pcDNA CM) or Wnt3a conditioned media (Wnt3a CM), together or without Sclerostin conditioned (SCL CM) media that was pre-incubated with either anti GFP-IgG2a (aGFP-IgG) or anti Sclerostin-IgG2a (aSCL-IgG) for 30 minutes at room temperature. (D) KS483 cells were stimulated with control conditioned media or Wnt3a conditioned media (Wnt3a CM), together or without Sclerostin conditioned media that was pre-incubated with either anti GFP-IgG2a (aGFP-IgG) or anti Sclerostin-IgG2a (aSCL-IgG) for 30 minutes at room temperature. RNA was isolated and AXIN2 mRNA levels were determined by qRT-PCR. (E) KS483 cells were stimulated with the indicated conditioned media, which were pre-incubated with the indicated Fab antibodies. Three days after stimulation cells were fixed and stained for alkaline phosphatase activity. Results were quantified using spectrometry (F).

Antibodies against Sclerostin were generated from the HuCAL GOLD antibody library [39] using recombinant murine Sclerostin in a solution panning (T.D. Mueller et al., manuscript in preparation). The Fab antibodies showing the best binding to Sclerostin were tested to see if they had an effect on binding of Sclerostin-GFP to 293 cells over-expressing LRP6. A single Fab antibody (AbD09097) inhibited binding of Sclerostin-GFP. Two other Fab antibodies (AbD09101 and AbD09172) although able to bind Sclerostin, were not able to neutralize the binding of Sclerostin-GFP to LRP6 (Fig. 5a and data not shown). A full human/mouse chimeric IgG2a antibody was created by cloning the binding epitopes of AbD09097 (VH and VL segments) into the pMORPH2_h/mIg vector [40]. KS483 cells were affinity labeled with [^125^I]Sclerostin that was pre-incubated for 30 minutes at room temperature with controls (Fc and aGFP-IgG) or Sclerostin neutralizing antibodies (AbD09097 and aSCL-IgG), followed by chemical crosslinking. Pre-incubation of [^125^I]Sclerostin with the neutralizing antibodies blocked the binding to LRP5/6 (Fig. 5b). This antibody was also used in a BAT-luc reporter assay in KS483 cells, with a human/mouse chimeric IgG2a antibody against GFP serving as a control. In the BAT-luc reporter assay the anti-Sclerostin antibody showed a significant rescue of the inhibition by Sclerostin on the Wnt3a induced BAT-luc activity (Fig. 5c). Furthermore this anti-Sclerostin antibody was also able to rescue the inhibition by Sclerostin on Wnt3a-induced AXIN2 mRNA expression (Fig. 5d) as well as Sclerostin-mediated inhibition of Alkaline Phosphatase (ALP) activity induced by Wnt3a (Fig. 5e). To minimize effects of Wnt3a on proliferation, KS483 cells were stimulated with Wnt3a after they had reached confluency, a state in which KS483 cells are contact inhibited in growth. Quantification of the Alkaline Phosphatase activity showed a significant rescue of around 50% (Fig. 5f). Thus AbD09097 has a potent neutralizing effect on Sclerostin-mediated inhibition of Wnt-induced signaling in osteoprogenitor cells.

## Discussion

During adulthood bone remodeling performs a pivotal role in maintaining bone mass and integrity. When the balance between bone forming osteoblasts and bone resorbing osteoclasts goes awry, this may lead to metabolic bone diseases that are characterized by either too low or high bone mass. Sclerosteosis is a rare high bone mass disorder that is caused by loss of Sclerostin expression [9], [10]. Sclerostin, which is expressed by osteocytes, is a negative regulator of canonical Wnt signaling. Here we show that canonical Wnt-induced responses in mesenchymal cells are critically dependent on LRP5 and LRP6. Sclerostin preferentially binds to LRP6, and to a significantly lesser extent to LRP5, and inhibits canonical Wnt-induced responses. In addition, we show that GFP-tagged Sclerostin is internalized, and subsequently degraded in a proteasome-dependent manner. Moreover, we describe a recombinant antibody selected by phage display that specifically interferes with binding of Sclerostin to the Wnt co-receptors LRP5 and LRP6 and stimulates osteoblast differentiation. Such antibodies may in the future be used for anabolic therapy of bone-related disorders as has been described for other Sclerostin antibodies [41]–[46].

Consistent with previous reports [16] we found that Sclerostin attenuated Wnt3a-induced responses, but full inhibition was never achieved. This observation might be explained with the preferential binding of Sclerostin to LRP6. Both receptors, LRP5 and 6, appear to mediate Wnt-induced responses as knockdown of only either one of both LRPs is insufficient to block Wnt signaling completely. In the presence of Sclerostin, Wnt/LRP6 signaling might be preferentially impaired, while LRP5-mediated signaling might be maintained, since knockdown of LRP6 did not lead to enhanced binding of Sclerostin to LRP5.

Compared to Sclerostin, DKK1 was found to be more potent in inhibiting Wnt3a-induced responses. DKK1 is known to inhibit both Wnt/LRP5 and Wnt/LRP6 responses [31], [32], [47]. Consistent with previous reports, we find that DKK1 inhibits LRP6 phosphorylation by Glycogen synthase kinase-3 (GSK-3) and Casein kinase 1 gamma (CK1γ) [48]. However, Sclerostin appears not to exert the same effect. This indicates that the mechanism of action of DKK1 and Sclerostin to inhibit Wnt3a activity is different despite the fact that both Wnt inhibitors target the same Wnt receptor component. This is fully in line with the differential binding domains of both antagonists for LRP5/6 [49]–[51]. Whereas Sclerostin interacts with the first propeller region, DKK1 interacts with the third and fourth propellers [50], [51]. Also, Sclerostin and DKK1 have differential modulatory and context dependent effects on different Wnt ligands. Sclerostin has been reported to potentiate Wnt reporter activity [21], however we have not observed this. This could be due to specific assay conditions, e.g. transfection versus exogenous addition of proteins, or reflecting different expression patterns of the components of the signaling complex. Since different Wnt family members have been shown to bind different domains of LRP5/6 [18], [21], [30], [52]. The internalization of Sclerostin-GFP in a Monodansylcadaverine-sensitive manner suggests that Sclerostin is internalized via a Clathrin-dependent route. DKK1 was previously shown to induce the internalization and recycling of LRP6 via a Clathrin-dependent mechanism [28], [36]. Internalized DKK1 was degraded and this could be inhibited by lysosome inhibitors [48]. We found that ectopic expression of LRP6 in 293T cells greatly enhanced Sclerostin-GFP binding and internalization. This suggests that Sclerostin’s high affinity receptor LRP6 mediates the internalization under these conditions. However, in non*-transfected KS483 cells shRNA-mediated* knockdown of LRP5/6 did not significantly affect the binding and internalization of Sclerostin-GFP, suggesting that Sclerostin-GFP may also be internalized in a LRP5/6 independent manner.

Similar as reported for the anti-Sclerostin antibody described by Veverka et al. [53], we found that our anti-Sclerostin antibody rescued the inhibitory effect of Sclerostin on Wnt3a-induced reporter activity. We extended the analysis and demonstrated that this anti-Sclerostin antibody antagonized Sclerostin-mediated inhibition on Wnt-induced AXIN2 expression and Wnt-3a-induced ALP activity. Moreover, we found that our Sclerostin-neutralizing antibody inhibited Sclerostin-GFP binding to cell surface associated LRP6 and its subsequent internalization and degradation.

So far, antibodies that have been shown to interfere with Sclerostin-mediated inhibition of canonical Wnt signaling have been shown to interact with loop 2 of sclerostin [53]. Structural studies analyzing the N-terminal propeller domain of LRP6 indeed revealed a binding site for a key sequence motif NXI present not only in Sclerostin (amino acid sequence NAI) but also in the members of the DKK family (DKK1: NAI; DKK2: NSI; DKK3: NNI) that is potentially involved in the specific recognition and binding of these Wnt inhibitors to the co-receptor LRP6 [51]. However, despite this potential overlapping binding site for Sclerostin and DKK family members [16], [51], our data strongly suggest that the Wnt3a inhibition mechanism by Sclerostin and DKKs differ on the extra- as well as on the intracellular site thereby likely disqualifying a simple competition of Wnt and Wnt inhibitors for a shared binding epitope on LRP5/6.

## Materials and Methods

### Antibodies

Anti-LRP5 (C-20) was purchased from Santa-Cruz, anti-LRP6 (1C10) from Abcam, phospho-LRP5/6 from Cell Signaling, anti-GAPDH (6C5) from Millipore and anti- FLAG-M2 from Sigma.

### Recombinant Wnts

Recombinant Wnt3a and Wnt9b were purchased from R&D systems.

### Recombinant Sclerostin Proteins

Recombinant full-length murine Sclerostin derived from expression in Baculovirus-transfected insect cells was prepared as described previously [35]. A Sclerostin-GFP fusion protein was obtained by inserting the cDNA encoding for eGFP at the C-terminal end of the above described expression construct for murine Sclerostin. As only an *Xho*I restriction site was available for the insertion of the GFP cDNA sequence, the expression vector pMKI_mSOST was modified by mutating the stop codon at the 3′ end of the Sclerostin cDNA to encode a glycine residue ahead of the *Xho*I restriction site. The cDNA of eGFP was then amplified from the bacterial expression vector pET28b_eGFP by PCR including a 5′ extension for an *Xho*I and an *Eco*RI restriction site and a 3′ extension encoding a stop codon, a *Xho*I and a *Sca*I restriction site. By analytical restriction analysis using a combination of *Bam*HI and *Eco*RI or *Bam*HI and *Sca*I the orientation of the GFP gene with respect to the Sclerostin cDNA was tested and the correctness of the cDNA sequence of the Sclerostin-GFP fusion was verified by DNA sequencing. Recombinant Baculovirus was obtained from cotransfection of the transfer vector pMKI_mSOST-GFP and linearized virus DNA (BAC-3000 virus kit, Novagen) into Sf9 insect cells. By using recombinant BAC-3000 virus production the yield of Sclerostin could be increased. Compared to BAC-2000 virus DNA, BAC-3000 DNA lacks the secreted Baculovirus genes *chiA* and *v-cath*, which encode for two secreted enzymes, a chitinase and a Cathepsin protease, both of which can lower the yield of recombinant protein. Virus selection and amplification was performed as suggested by the manufacturer. For protein production, semi-adherent growing TriEX insect cells (Novagen) were transfected with recombinant virus using a MOI of 5. Cell supernatant was harvested 96 h post transfection and clarified by centrifugation. After dialysis against 50 mM sodium phosphate pH 8.3, 300 mM NaCl, 10 mM Imidazole Sclerostin-GFP fusion protein was isolated by metal affinity chromatography using a Ni-NTA Perfect Pro column (5 Prime) and eluting the protein with dialysis buffer containing 250 mM Imidazole. Protein-containing fractions were pooled and dialyzed against 10 mM HEPES pH 7 u.4, 3.4 mM EDTA, 150 mM NaCl. For final purification a cation exchange chromatography was performed employing a CM-Sepharose HiTrap column (GE healthcare) and a linear gradient from 150 mM to 1 M NaCl in 10 mM HEPES pH 7.4. Fractions were analyzed by SDS-PAGE and pooled accordingly. Sclerostin-GFP was finally dialyzed against PBS, two times diluted with 86% Glycerol for cryoprotection and stored at −20°C until further use.

GFP protein for control studies was obtained from bacterial expression using the expression vector pET28b_eGFP encoding eGFP with an N-terminal His_6_-tag and a subsequent thrombin cleavage site. Isolation and purification of recombinant GFP via metal affinity and a subsequent cation exchange chromatography followed the protocol described above.

### Cell Culture

COS, 293 and C2C12 (ATCC) cells were cultured in Dulbecco’s modified Eagle’s medium with high glucose (GIBCO) containing 10% FBS (GIBCO). C3H10T1/2 (ATCC) and KS483 [54] cells were cultured in αMEM (GIBCO) containing 10% FBS. ATDC5 [55] cells were cultured in DMEM/F12 (GIBCO) containing 10% FBS (GIBCO). All cells were cultured at at 37°C with 5% CO_2_.

### Transcriptional Reporter Assay

Cells were seeded in 24-well plates and transiently transfected for 4 hours with the different expression plasmids, a β-Galactosidase expression plasmid and the BAT-luc reporter construct [56] using Lipofectamine reagent (Invitrogen) according to the manufacturer’s protocol. After two days the cells were serum-starved for 8 hours and subsequently stimulated for 16 hours with the indicated conditioned medium. Cells were washed, lysed and activity of Luciferase and β-Galactosidase, which served as a control to correct for transfection efficiency, was determined. Each transfection was carried out in triplicate and representative experiments are shown.

### RNA Isolation and Quantitative Real-time PCR

Total RNA was isolated using the Nucleospin RNA II kit (Macherery-Nagel) according to the manufacturer’s instructions. RT-PCR was performed using the RevertAid H Minus First Strand cDNA synthesis kit (Fermentas) following the manufacturer’s recommendation. Expression of mouse LRP5, LRP6, AXIN2 and GAPDH was analyzed in triplicate. PCRs were performed using SYBR GREEN (Roche) on the StepOne Plus real-time PCR system (Applied Biosystems). Gene transcription levels were determined with the comparative ΔCt method using GAPDH as a reference. PCR primers; mouse LRP5 forward 5′TGG GAC TCA AAG CCG TGA AT3′, mouse LRP5 reverse 5′TGG CTG CAC CCT CCA TTT3′, mouse LRP6 forward 5′AGA TCC ATC AAG TGG GTT CAT GTA3′, mouse LRP6 reverse 5′AAG CGA CTT GAG CCA TCC AT3′, mouse AXIN2 forward 5′GGT TCC GGC TAT GTC TTT GC3′, mouse AXIN2 reverse 5′CAG TGC GTC GCT GGA TAA CTC3, mouse GAPDH forward 5′AAC TTT GGC ATT GTG GAA GG3′, mouse GAPDH reverse 5′ACA CAT TGG GGG TAG GAA CA3′.

### Western Blot Analysis

Cells were seeded in 6-well plates and allowed to grow to confluence. Cells were washed with PBS and lysed in SDS sample buffer. Samples were boiled for 5 minutes and subjected to SDS-PAGE and Western blotting.

### [^125^I]Sclerostin Binding Assay

Iodination of recombinant murine full-length Sclerostin was performed according to the chloramine T method and cells were subsequently affinity-labeled with the radioactive ligand as described before [57,58]. In brief, cells were incubated on ice for 3 hours with the radioactive ligand. After incubation, cells were washed and crosslinking was performed using 0.27 mM Disuccinimidyl suberate (DSS, Pierce) and 0.09 mM Bis(sulfosuccinimidyl)suberate (BS3, Pierce) for 15 minutes. Cells were washed, scraped and lysed. Lysates were incubated with the respective antisera for 2.5 hours and immune complexes were precipitated by adding protein A Sepharose (Amersham Biosciences). Samples were washed, boiled in SDS sample buffer and subjected to SDS-PAGE. Gels were dried and scanned with the STORM imaging system (Amersham Biosciences). Bands were quantified using ImageQuant TL v2003.03 software (Amersham Biosciences).

### Sclerostin-GFP Internalization Assay

Cells were grown on glass slides. Cells were cooled down on ice for 0.5 h and subsequently incubated for 2.5 h on ice with 500 ng/ml Sclerostin-GFP. Next the cells were placed in a 37°C incubator for the indicated time. After washing one time with PBS the cells were fixed with 3% paraformaldehyde in phosphate-buffer. Slides were mounted and confocal microscopy pictures were taken (Leica TCS SL).

### Statistical Analysis

The unpaired two-tailed Student’s t test was used to determine the significance of differences between means. All relevant comparisons were significantly different (P<0.05), unless otherwise indicated. Experiments were performed at least three times and representative results are shown.

## Supporting Information

Figure S1 (TIF)

Figure S2 (TIF)

## References

[1] Olson BR (2006) Bone embryology. In: Favus MJ, editor. Primer on the Metabolic Bone Diseases And Disorders of Mineral Metabolism. Washington DC: American society for bone and mineral research. 2–6.

[2] Marie PJ (2012) Signaling pathways affecting skeletal health. Current osteoporosis reports 10: 190–198. 22711369 10.1007/s11914-012-0109-0

[3] Rachner TD, Khosla S, Hofbauer LC (2011) Osteoporosis: now and the future. Lancet 377: 1276–1287. 21450337 10.1016/S0140-6736(10)62349-5 PMC3555696

[4] Kawai M, Mödder UI, Khosla S, Rosen CJ (2011) Emerging therapeutic opportunities for skeletal restoration. Nature reviews Drug discovery 10: 141–156. 21283108 10.1038/nrd3299 PMC3135105

[5] Baron R, Hesse E (2012) Update on bone anabolics in osteoporosis treatment: rationale, current status, and perspectives. The Journal of clinical endocrinology and metabolism 97: 311–325. 22238383 10.1210/jc.2011-2332 PMC3275361

[6] Janssens K, Van Hul W (2002) Molecular genetics of too much bone. Human molecular genetics 11: 2385–2393. 12351574 10.1093/hmg/11.20.2385

[7] Kaplan FS, Pignolo RJ, Shore EM (n.d.) The FOP metamorphogene encodes a novel type I receptor that dysregulates BMP signaling. Cytokine & growth factor reviews 20: 399–407. 10.1016/j.cytogfr.2009.10.006 PMC3515059 19896889

[8] Lewiecki EM (2011) New targets for intervention in the treatment of postmenopausal osteoporosis. Nature reviews Rheumatology 7: 631–638. 21931340 10.1038/nrrheum.2011.130

[9] Balemans W, Van Hul W (2004) Identification of the disease-causing gene in sclerosteosis–discovery of a novel bone anabolic target? Journal of musculoskeletal & neuronal interactions 4: 139–142. 15615113

[10] van Bezooijen RL, ten Dijke P, Papapoulos SE, Löwik CWGM (2005) SOST/sclerostin, an osteocyte-derived negative regulator of bone formation. Cytokine & growth factor reviews 16: 319–327. 15869900 10.1016/j.cytogfr.2005.02.005

[11] Balemans W, Ebeling M, Patel N, Van Hul E, Olson P, et al (2001) Increased bone density in sclerosteosis is due to the deficiency of a novel secreted protein (SOST). Human molecular genetics 10: 537–543. 11181578 10.1093/hmg/10.5.537

[12] Brunkow ME, Gardner JC, Van Ness J, Paeper BW, Kovacevich BR, et al (2001) Bone dysplasia sclerosteosis results from loss of the SOST gene product, a novel cystine knot-containing protein. American journal of human genetics 68: 577–589. 11179006 10.1086/318811 PMC1274471

[13] Li X, Ominsky MS, Niu Q-T, Sun N, Daugherty B, et al (2008) Targeted deletion of the sclerostin gene in mice results in increased bone formation and bone strength. Journal of bone and mineral research: the official journal of the American Society for Bone and Mineral Research 23: 860–869. 10.1359/jbmr.080216 18269310

[14] Krause C, Korchynskyi O, de Rooij K, Weidauer SE, de Gorter DJJ, et al (2010) Distinct modes of inhibition by sclerostin on bone morphogenetic protein and Wnt signaling pathways. The Journal of biological chemistry 285: 41614–41626. 20952383 10.1074/jbc.M110.153890 PMC3009889

[15] Winkler DG, Sutherland MK, Geoghegan JC, Yu C, Hayes T, et al (2003) Osteocyte control of bone formation via sclerostin, a novel BMP antagonist. The EMBO journal 22: 6267–6276. 14633986 10.1093/emboj/cdg599 PMC291840

[16] Li X, Zhang Y, Kang H, Liu W, Liu P, et al (2005) Sclerostin binds to LRP5/6 and antagonizes canonical Wnt signaling. The Journal of biological chemistry 280: 19883–19887. 15778503 10.1074/jbc.M413274200

[17] Semënov M, Tamai K, He X (2005) SOST is a ligand for LRP5/LRP6 and a Wnt signaling inhibitor. The Journal of biological chemistry 280: 26770–26775. 15908424 10.1074/jbc.M504308200

[18] van Bezooijen RL, Svensson JP, Eefting D, Visser A, van der Horst G, et al (2007) Wnt but not BMP signaling is involved in the inhibitory action of sclerostin on BMP-stimulated bone formation. Journal of bone and mineral research: the official journal of the American Society for Bone and Mineral Research 22: 19–28. 10.1359/jbmr.061002 17032150

[19] Tamai K, Semenov M, Kato Y, Spokony R, Liu C, et al (2000) LDL-receptor-related proteins in Wnt signal transduction. Nature 407: 530–535. 11029007 10.1038/35035117

[20] Pinson KI, Brennan J, Monkley S, Avery BJ, Skarnes WC (2000) An LDL-receptor-related protein mediates Wnt signalling in mice. Nature 407: 535–538. 11029008 10.1038/35035124

[21] Ettenberg SA, Charlat O, Daley MP, Liu S, Vincent KJ, et al (2010) Inhibition of tumorigenesis driven by different Wnt proteins requires blockade of distinct ligand-binding regions by LRP6 antibodies. Proceedings of the National Academy of Sciences of the United States of America 107: 15473–15478. 20713706 10.1073/pnas.1007428107 PMC2932603

[22] Boyden LM, Mao J, Belsky J, Mitzner L, Farhi A, et al (2002) High bone density due to a mutation in LDL-receptor-related protein 5. The New England journal of medicine 346: 1513–1521. 12015390 10.1056/NEJMoa013444

[23] Little RD, Carulli JP, Del Mastro RG, Dupuis J, Osborne M, et al (2002) A mutation in the LDL receptor-related protein 5 gene results in the autosomal dominant high-bone-mass trait. American journal of human genetics 70: 11–19. 11741193 10.1086/338450 PMC419982

[24] Ellies DL, Viviano B, McCarthy J, Rey J-P, Itasaki N, et al (2006) Bone density ligand, Sclerostin, directly interacts with LRP5 but not LRP5G171V to modulate Wnt activity. Journal of bone and mineral research: the official journal of the American Society for Bone and Mineral Research 21: 1738–1749. 10.1359/jbmr.060810 17002572

[25] Leupin O, Piters E, Halleux C, Hu S, Kramer I, et al (2011) Bone overgrowth-associated mutations in the LRP4 gene impair sclerostin facilitator function. The Journal of biological chemistry 286: 19489–19500. 26. Clevers H, Nusse R (2012) Wnt/β-Catenin Signaling and Disease. Cell 149: 1192–1205. 10.1074/jbc.M110.190330 PMC3103328 21471202

[26] Jho E, Zhang T, Domon C, Joo C-K, Freund J-N, et al (2002) Wnt/beta-catenin/Tcf signaling induces the transcription of Axin2, a negative regulator of the signaling pathway. Molecular and cellular biology 22: 1172–1183. 11809808 10.1128/MCB.22.4.1172-1183.2002 PMC134648

[27] Mao B, Wu W, Davidson G, Marhold J, Li M, et al (2002) Kremen proteins are Dickkopf receptors that regulate Wnt/beta-catenin signalling. Nature 417: 664–667. 12050670 10.1038/nature756

[28] Brott BK, Sokol SY (2002) Regulation of Wnt/LRP signaling by distinct domains of Dickkopf proteins. Molecular and cellular biology 22: 6100–6110. 12167704 10.1128/MCB.22.17.6100-6110.2002 PMC133995

[29] Bourhis E, Tam C, Franke Y, Bazan JF, Ernst J, et al (2010) Reconstitution of a frizzled8.Wnt3a.LRP6 signaling complex reveals multiple Wnt and Dkk1 binding sites on LRP6. The Journal of biological chemistry 285: 9172–9179. 20093360 10.1074/jbc.M109.092130 PMC2838336

[30] Mao B, Wu W, Li Y, Hoppe D, Stannek P, et al (2001) LDL-receptor-related protein 6 is a receptor for Dickkopf proteins. Nature 411: 321–325. 11357136 10.1038/35077108

[31] Semënov MV, Tamai K, Brott BK, Kühl M, Sokol S, et al (2001) Head inducer Dickkopf-1 is a ligand for Wnt coreceptor LRP6. Current biology: CB 11: 951–961. 11448771 10.1016/s0960-9822(01)00290-1

[32] Zhang J, Zhang X, Zhang L, Zhou F, van Dinther M, et al (2012) LRP8 mediates Wnt/β-catenin signaling and controls osteoblast differentiation. Journal of bone and mineral research: the official journal of the American Society for Bone and Mineral Research 27: 2065–2074. 10.1002/jbmr.1661 22589174

[33] Niehrs C, Shen J (2010) Regulation of Lrp6 phosphorylation. Cellular and molecular life sciences: CMLS 67: 2551–2562. 20229235 10.1007/s00018-010-0329-3 PMC11115861

[34] Weidauer SE, Schmieder P, Beerbaum M, Schmitz W, Oschkinat H, et al (2009) NMR structure of the Wnt modulator protein Sclerostin. Biochemical and biophysical research communications 380: 160–165. 19166819 10.1016/j.bbrc.2009.01.062

[35] Yamamoto H, Sakane H, Michiue T, Kikuchi A (2008) Wnt3a and Dkk1 regulate distinct internalization pathways of LRP6 to tune the activation of beta-catenin signaling. 10.1016/j.devcel.2008.04.015 18606139

[36] Schlegel R, Dickson RB, Willingham MC, Pastan IH (1982) Amantadine and dansylcadaverine inhibit vesicular stomatitis virus uptake and receptor-mediated endocytosis of alpha 2-macroglobulin. Proceedings of the National Academy of Sciences of the United States of America 79: 2291–2295. 6179094 10.1073/pnas.79.7.2291 PMC346178

[37] Rothberg KG, Heuser JE, Donzell WC, Ying YS, Glenney JR, et al (1992) Caveolin, a protein component of caveolae membrane coats. Cell 68: 673–682. 1739974 10.1016/0092-8674(92)90143-z

[38] Rothe C, Urlinger S, Löhning C, Prassler J, Stark Y, et al (2008) The human combinatorial antibody library HuCAL GOLD combines diversification of all six CDRs according to the natural immune system with a novel display method for efficient selection of high-affinity antibodies. Journal of molecular biology 376: 1182–1200. 18191144 10.1016/j.jmb.2007.12.018

[39] Ostendorp R, Frisch C, Urban M (2004) Generation, engineering and production of human antibodies using HuCAL. In: Subramanian G, editor. Antibodies Volume 2: Novel Technologies and Therapeutic Use. New York: Kluwer Academic/Plenum Publishers. 13–52.

[40] Li X, Ominsky MS, Warmington KS, Morony S, Gong J, et al (2009) Sclerostin antibody treatment increases bone formation, bone mass, and bone strength in a rat model of postmenopausal osteoporosis. Journal of bone and mineral research: the official journal of the American Society for Bone and Mineral Research 24: 578–588. 10.1359/jbmr.081206 19049336

[41] Ominsky MS, Vlasseros F, Jolette J, Smith SY, Stouch B, et al (2010) Two doses of sclerostin antibody in cynomolgus monkeys increases bone formation, bone mineral density, and bone strength. Journal of bone and mineral research: the official journal of the American Society for Bone and Mineral Research 25: 948–959. 10.1002/jbmr.14 20200929

[42] Ominsky MS, Li C, Li X, Tan HL, Lee E, et al (2011) Inhibition of sclerostin by monoclonal antibody enhances bone healing and improves bone density and strength of nonfractured bones. Journal of bone and mineral research: the official journal of the American Society for Bone and Mineral Research 26: 1012–1021. 10.1002/jbmr.307 21542004

[43] Padhi D, Jang G, Stouch B, Fang L, Posvar E (2011) Single-dose, placebo-controlled, randomized study of AMG 785, a sclerostin monoclonal antibody. Journal of bone and mineral research: the official journal of the American Society for Bone and Mineral Research 26: 19–26. 10.1002/jbmr.173 20593411

[44] Virdi AS, Liu M, Sena K, Maletich J, McNulty M, et al (2012) Sclerostin antibody increases bone volume and enhances implant fixation in a rat model. The Journal of bone and joint surgery American volume 94: 1670–1680. 22992878 10.2106/JBJS.K.00344 PMC3444952

[45] McDonald MM, Morse A, Mikulec K, Peacock L, Yu N, et al (2012) Inhibition of sclerostin by systemic treatment with sclerostin antibody enhances healing of proximal tibial defects in ovariectomized rats. Journal of orthopaedic research: official publication of the Orthopaedic Research Society 30: 1541–1548. 22457198 10.1002/jor.22109

[46] Bafico A, Liu G, Yaniv A, Gazit A, Aaronson SA (2001) Novel mechanism of Wnt signalling inhibition mediated by Dickkopf-1 interaction with LRP6/Arrow. Nature cell biology 3: 683–686. 11433302 10.1038/35083081

[47] Sakane H, Yamamoto H, Kikuchi A (2010) LRP6 is internalized by Dkk1 to suppress its phosphorylation in the lipid raft and is recycled for reuse. Journal of cell science 123: 360– 10.1242/jcs.058008 20053636

[48] Ahn VE, Chu ML-H, Choi H-J, Tran D, Abo A, et al (2011) Structural basis of Wnt signaling inhibition by Dickkopf binding to LRP5/6. Developmental cell 21: 862–873. 22000856 10.1016/j.devcel.2011.09.003 PMC3215855

[49] Cheng Z, Biechele T, Wei Z, Morrone S, Moon RT, et al (2011) Crystal structures of the extracellular domain of LRP6 and its complex with DKK1. Nature structural & molecular biology 18: 1204–1210. 10.1038/nsmb.2139 PMC3249237 21984209

[50] Bourhis E, Wang W, Tam C, Hwang J, Zhang Y, et al (2011) Wnt antagonists bind through a short peptide to the first β-propeller domain of LRP5/6. Structure (London, England: 1993) 19: 1433–1442. 10.1016/j.str.2011.07.005 21944579

[51] Gong Y, Bourhis E, Chiu C, Stawicki S, DeAlmeida VI, et al (2010) Wnt isoform-specific interactions with coreceptor specify inhibition or potentiation of signaling by LRP6 antibodies. PloS one 5: e12682. 10.1371/journal.pone.0012682 PMC2938341 20856934

[52] Veverka V, Henry AJ, Slocombe PM, Ventom A, Mulloy B, et al (2009) Characterization of the structural features and interactions of sclerostin: molecular insight into a key regulator of Wnt-mediated bone formation. The Journal of biological chemistry 284: 10890–10900. 19208630 10.1074/jbc.M807994200 PMC2667775

[53] Yamashita T, Ishii H, Shimoda K, Sampath TK, Katagiri T, et al (1996) Subcloning of three osteoblastic cell lines with distinct differentiation phenotypes from the mouse osteoblastic cell line KS-4. Bone 19: 429–436. 8922640 10.1016/s8756-3282(96)00255-4

[54] Atsumi T, Miwa Y, Kimata K, Ikawa Y (1990) A chondrogenic cell line derived from a differentiating culture of AT805 teratocarcinoma cells. Cell differentiation and development: the official journal of the International Society of Developmental Biologists 30: 109–116. 2201423 10.1016/0922-3371(90)90079-c

[55] Maretto S, Cordenonsi M, Dupont S, Braghetta P, Broccoli V, et al (2003) Mapping Wnt/beta-catenin signaling during mouse development and in colorectal tumors. Proceedings of the National Academy of Sciences of the United States of America 100: 3299–3304. 12626757 10.1073/pnas.0434590100 PMC152286

[56] Frolik CA, Wakefield LM, Smith DM, Sporn MB (1984) Characterization of a membrane receptor for transforming growth factor-beta in normal rat kidney fibroblasts. The Journal of biological chemistry 259: 10995–11000. 6088525

[57] Yamashita H, ten Dijke P, Huylebroeck D, Sampath TK, Andries M, et al (1995) Osteogenic protein-1 binds to activin type II receptors and induces certain activin-like effects. The Journal of cell biology 130: 217–226. 7790373 10.1083/jcb.130.1.217 PMC2120513

