# Supplementary figures and images for: Anti-Sclerostin Antibody Inhibits Internalization of Sclerostin and Sclerostin-Mediated Antagonism of Wnt/LRP6 Signaling
Source: PLoS One. 2013 Apr 29;8(4):e62295. doi: 10.1371/journal.pone.0062295 (PMC3639248; doi:10.1371/journal.pone.0062295)

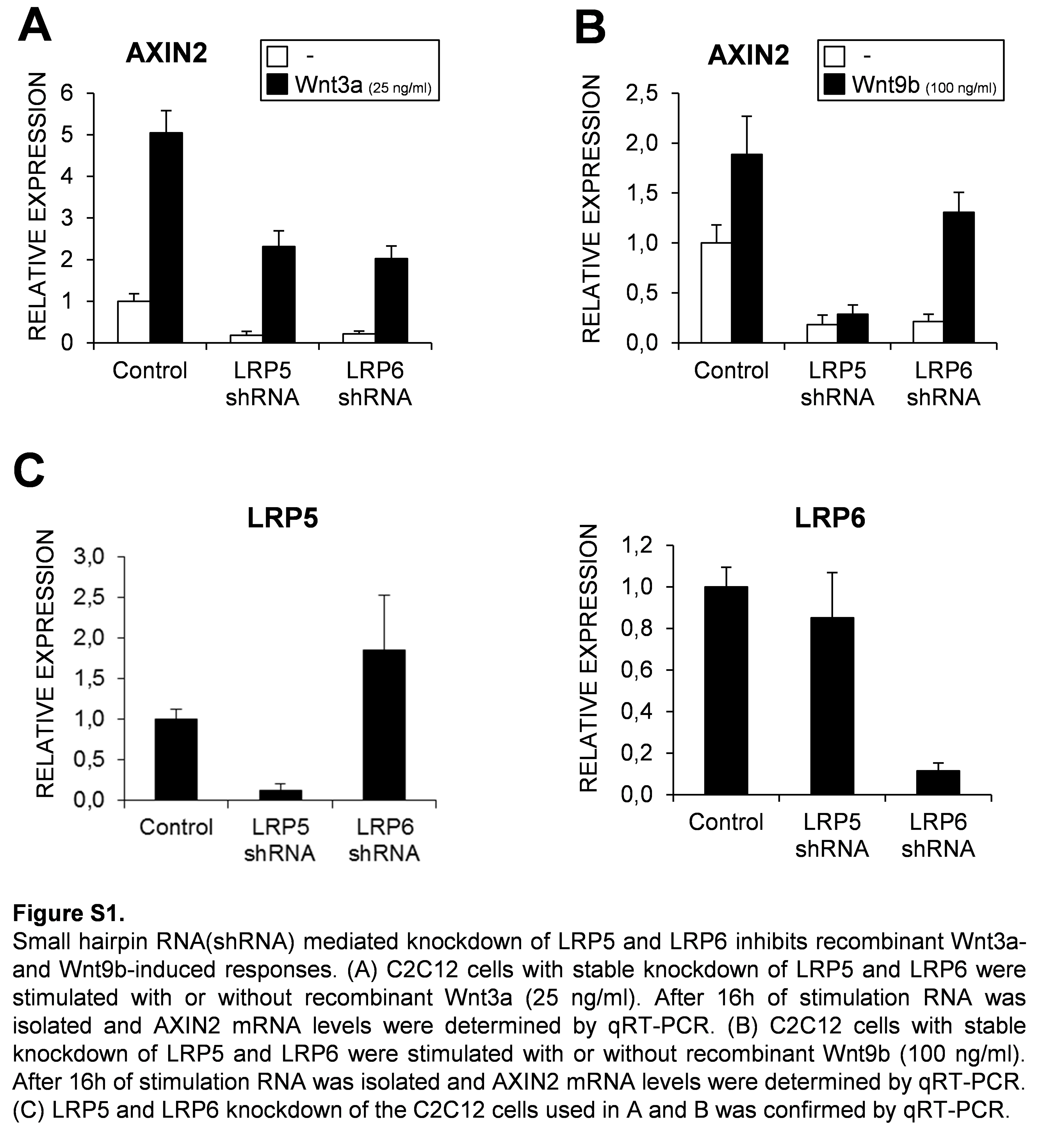

Supplement: Figure S1 — (TIF) [file pone.0062295.s001.tif]

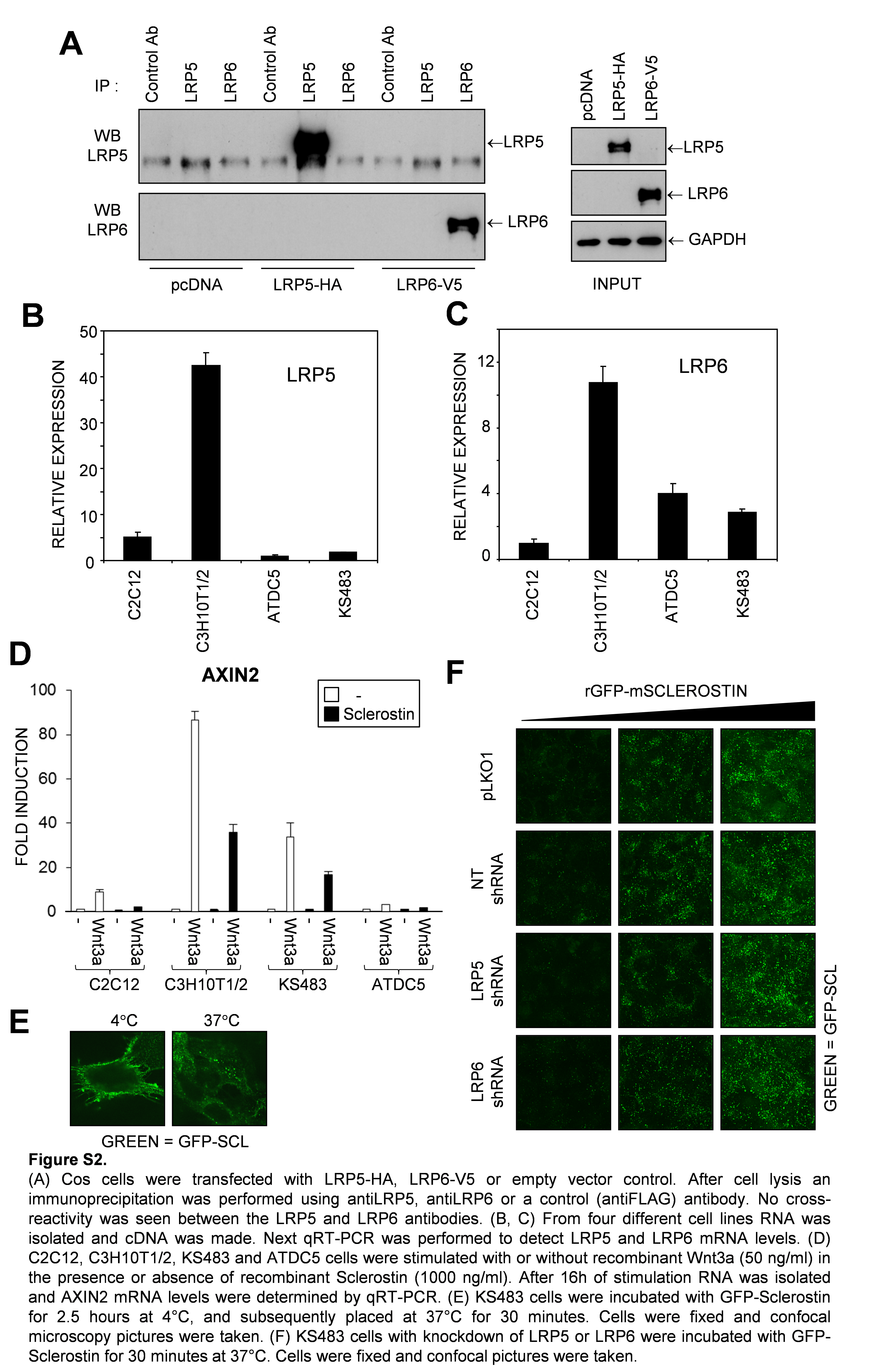

Supplement: Figure S2 — (TIF) [file pone.0062295.s002.tif]
